# Triglyceride-glucose-Body Mass Index is linked to papillary thyroid carcinoma odds in China: a retrospective study

**DOI:** 10.3389/fendo.2026.1766186

**Published:** 2026-03-30

**Authors:** Guoqing Li, Qingyu Ren, Jiaqin Zhou, Hui Zhou, Yibo Wu, Ling Chen, Kai Jiang

**Affiliations:** 1 Affiliated Hospital of Jiangnan University, Jiangnan University, Wuxi, Jiangsu, China; 2 Human Reproductive and Genetic Center, Affiliated Hospital of Jiangnan University, Wuxi, Jiangsu, China; 3 Department of General Surgery, Affiliated Hospital of Jiangnan University, Wuxi, Jiangsu, China

**Keywords:** biomarker, BMI - body mass index, insulin resistance, papillary thyroid carcinoma, triglyceride glucose-body mass index

## Abstract

**Background:**

The incidence of thyroid cancer is gradually increasing year by year, with papillary thyroid carcinoma being the predominant type of thyroid cancer. Exploring the risk factors for papillary thyroid carcinoma is crucial for predicting and treating papillary thyroid carcinoma. This research investigated the correlation between the odds of papillary thyroid carcinoma (PTC) and the triglyceride-glucose-Body Mass Index (TyG-BMI) among the Chinese population.

**Methods:**

The study enrolled 213 individuals diagnosed with thyroid nodule (TN) and 325 individuals with papillary thyroid carcinoma (PTC) to examine the association between the TyG-BMI index and the odds of papillary thyroid carcinoma. We used the restricted cubic spline (RCS) curve to explore the non-linear relationship between TyG - BMI and papillary thyroid carcinoma. The logistic regression model was employed to assess the odds relationship between TyG - BMI and papillary thyroid carcinoma. All methods are carried out in accordance with the relevant guidelines and regulations.

**Result:**

TyG-BMI (triglyceride glucose-body mass index) is positively correlated with the odds of papillary thyroid carcinoma (PTC) in a dose - response manner: for each 1-unit increase in this continuous variable, the odds ratio (OR) is approximately 1.02 (P<0.001). The OR value in the group with TyG - BMI higher than the median is 1.79 (95%CI: 1.13-2.83, P = 0.013), and the statistical significance remains after Holm-Bonferroni correction. Receiver operating characteristic (ROC) curve analysis shows that the area under the curve (AUC) of TyG - BMI for diagnosing PTC is 0.64, which is superior to that of BMI alone (AUC = 0.61).

**Conclusion:**

It was found that an increased TyG - BMI index is correlated with a higher likelihood of papillary thyroid carcinoma (PTC), but larger - scale studies are needed to verify our founding.

## Background

Globally, the incidence of thyroid cancer has been on the rise since the 1990s, with a growth rate that exceeds that of any other malignant tumor (1). It is estimated that the age - standardized incidence rate of thyroid cancer worldwide in 2020 was approximately 10.1 cases per 100,000 females and 3.1 cases per 100,000 males, with mortality rates at 0.5 cases per 100,000 females and 0.3 cases per 100,000 males (1). The incidence rate among females is more than 15 times that of males, with the highest rates found in North America and East Asia (2). Papillary thyroid carcinoma (PTC) is the most common type of thyroid cancer, accounting for 80% to 85% of all thyroid cancer cases (3). Recognizing controllable risk factors and individuals at high risk is essential for mitigating the societal effects of papillary thyroid carcinoma (4). Even with considerable progress in papillary thyroid carcinoma detection, diagnosis, treatment, and surveillance for recurrence over the past several decades, the occurrence of papillary thyroid carcinoma remains on the rise (5). Therefore, it is crucial to develop practical and reliable non-invasive markers to identify high-risk populations, which will aid in risk stratification and reduce the incidence and mortality rates.

Metabolic syndrome (MetS), defined by a cluster of biological abnormalities including obesity, abnormal lipid levels, impaired glucose regulation, and high blood pressure, is closely associated with a variety of diseases, cancer among them (6, 7). For papillary thyroid carcinoma specifically, MetS has been associated with an increased risk of developing more aggressive forms of the disease and a less favorable prognosis (8). Insulin resistance (IR), an important element of MetS, plays a vital role in the development of papillary thyroid carcinoma (9). Triglyceride Glucose - Body Mass Index (TyG - BMI) is an emerging indicator used to assess MetS. Studies have shown that TyG - BMI is significantly correlated with MetS, especially in individuals without diabetes. It is considered a simple and clinically useful surrogate marker for MetS (10). The TyG - BMI index, which integrates both body mass index (BMI) and triglyceride glucose index (TyG), has been found to be more advantageous than TyG alone in assessing MetS and predicting various health outcomes (11–13). A study also demonstrated that TyG - BMI has a causal association with the risk of MetS, particularly in young, middle - aged, and non - obese populations, where this independent association is more evident (14). In addition, there are studies that have explored the relationship between TyG - BMI and the risk of MetS, finding a significant nonlinear relationship between them. These studies suggest that TyG - BMI may be a useful indicator for the early identification of MetS, especially in the prevention and management of diabetes (15). It has been shown to be practical, potent, and reproducible, with high sensitivity and specificity in the detection (14, 16). Moreover, multiple studies have demonstrated that TyG - BMI is more effective than Homeostasis Model Assessment (HOMA) in predicting metabolic syndrome (17, 18).

The epidemiological data of papillary thyroid carcinoma in China shows that the incidence rate has been on the rise in recent years. Thyroid cancer has become the seventh most common malignant tumor globally, and in China, its age-specific incidence rate ranks third among all malignant tumors (19). In light of this increasing trend, it is necessary to further investigate the factors affecting the incidence of papillary thyroid carcinoma and identify simple indicators for odds stratification of patients. Currently, the TyG - BMI index has been proven to be associated with several types of cancers, but no studies have explored its relationship with papillary thyroid carcinoma (20). So, the study aimed to look at how the TyG - BMI index is related to papillary thyroid carcinoma in the Chinese population, filling an important research gap.

## Methods

### Study design and subjects

**Figure 1:**
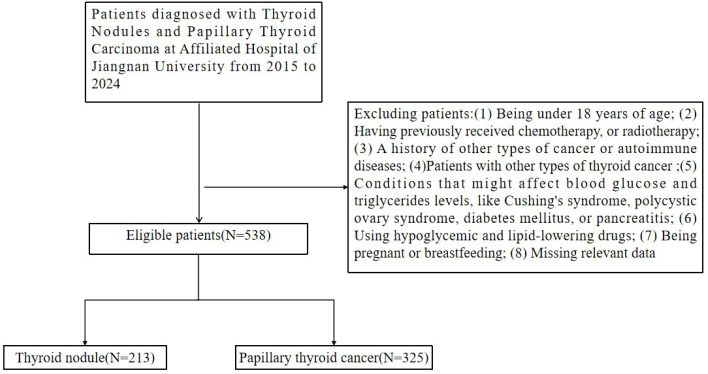
Flow chart of study subjects.

This study is a retrospective study. A review of medical records was performed for 538 papillary thyroid carcinoma and thyroid nodule patients who underwent treatment by the Thyroid Surgery Department at Jiangnan University Affiliated Hospital between January 2015 and December 2024. The ethics committee of Jiangnan University Affiliated Hospital granted approval for this study (approval number: JSMS0124000121), and the requirement for informed consent was waived because the data used was anonymized. All methods were carried out in accordance with the relevant guidelines and regulations, including the Declaration of Helsinki. The information of all patients was collected before the first operation. The blood samples of all patients are collected between 6 and 8 in the morning after fasting for 8 hours. However, patients were excluded from the study if they met any of the following criteria: (1) Being under 18 years of age; (2) Having previously received chemotherapy, or radiotherapy; (3) A history of other types of cancer or autoimmune diseases; (4)Patients with other types of thyroid cancer; (5) Conditions that might affect blood glucose and triglycerides levels, like Cushing’s syndrome, polycystic ovary syndrome, diabetes mellitus, or pancreatitis; (6) Using hypoglycemic and lipid-lowering drugs; (7) Being pregnant or breastfeeding; (8) Missing relevant data. Ultimately, the analysis considered a cohort comprising 538 individuals, including 213 cases of thyroid nodule and 325 cases of thyroid papillary cancer. **Figure 1** illustrates the data inclusion flow of this study.

### Data collection

In this study, personal information was processed using the “de-identification + Safe Harbor standard” approach: eighteen direct identifiers including names, national identification numbers, addresses, and phone numbers were removed; dates of birth were truncated to the year only, and medical visit dates were shifted to the corresponding quarter; continuous variables (such as age and test values) were binned based on clinical cut-off points, with extreme values subjected to top coding; free-text fields were manually reviewed to eliminate descriptive terms that might disclose personal identities, and then the Secure Hash Algorithm 256-bit (SHA-256) was used to generate irreversible unique codes to replace the original medical record numbers. The processed dataset has been stripped of the “identifiable information” as defined in the Personal Information Protection Law of the People’s Republic of China (PIPL). A third-party privacy risk assessment confirmed that the re-identification probability is less than 0.05, which complies with the Safe Harbor provisions under the Health Insurance Portability and Accountability Act (HIPAA).

Information on participants’ gender, age, weight, height, hypertension status was gathered from the hospital information system. Furthermore, the lab information system supplied data on the serum levels of total cholesterol, triglycerides, high-density lipoprotein cholesterol, low-density lipoprotein cholesterol, fasting blood glucose, leukocyte count, thyroid-stimulating hormone (TSH), thyroglobulin, anti-thyroglobulin antibody levels and thyroid peroxidase antibody.

### Laboratory analysis

The hospital’s clinical laboratory utilized an AU5800 chemistry analyzer from Beckman to measure the concentrations of total cholesterol, triglycerides, high - density lipoprotein cholesterol, low - density lipoprotein cholesterol, leukocyte count and fasting blood glucose. The white blood cell count is examined using the Mindray semi-automatic biochemistry analyzer BA - 88A. All test reagents are original factory reagents. Blood samples from all patients were collected after fasting for 8 hours, between 6 and 8 AM in the morning, and immediately sent for testing after collection. The analyzer underwent routine daily quality assessments and annual calibrations to ensure its accuracy and reliability. All procedures were conducted in accordance with the standard operating protocols of the analyzer to guarantee consistency and precision. The normal reference ranges for the related substances involved in this study are presented in **Table 1**.

**Table 1:** The normal reference ranges for the relevant substances in this study.

Project name	Reference range
Albumin	40–55 g/L
Glucose	3.9 - 6.1 mmol/L
Total Cholesterol	3.5 - 5.18 mmol/L
Triglycerides	0 - 1.7 mmol/L
High - Density Lipoprotein Cholesterol	1.04 - 1.55 mmol/L
Low - Density Lipoprotein Cholesterol	0 - 3.37 mmol/L
Leukocyte	(4 - 10) *109/L
Thyroid - Stimulating Hormone	0.3 - 0.6 mIU/L
Thyroglobulin	0.9–54 ng/mL
Thyroglobulin Antibody	5–100 IU/mL
Anti - Thyroid Peroxidase Antibody	1–16 IU/mL

### Definition of index

The computation of the indexes was demonstrated using a formula:


$$\text{Body mass index }(\text{BMI})\;=\text{ weight }(\text{kg})/\text{height squared }{(\text{m}}^{2}).$$

TyG - BMI index = Ln [TG (mg/dl) × BG (mg/dl)/2] × BMI

(21)

### Statistical analysis

During the course of this research, the R 4.3.0 program was employed for statistical analysis. Data that were continuously distributed and followed a normal distribution are depicted as the Mean ± Standard Deviation, with the t-test for two independent samples used to assess differences between the two groups. For continuously distributed data that were not normally distributed, the Median (Q_1_, Q_3_) is used for representation, and the Mann - Whitney U test is applied for comparing two independent samples. Categorical data are presented as frequencies (n (%)), with the chi-square test or Fisher’s exact test used for comparisons. Multivariable Logistic Regression Model was conducted for analyses, and a significance level of P < 0.05 was considered to be statistically significant. The ROC curve was used to evaluate the diagnostic efficacy of the Tyg - BMI index for papillary thyroid carcinoma. Moreover, the study sought to explore the potential non-linearity in the dose-response relationship and to evaluate the correlation between the TyG - BMI index and the odds of papillary thyroid carcinoma development.

## Results

### Basic features of the enrolled participants

**Table 2** displayed the demographic characteristics of the participants involved in the study. There were 538 people in this study, of whom 213 were TN (accounting for 39.59%) and 325 were PTC (accounting for 60.41%). Age, height, weight, BMI, WBC, TgAb, THG, TSH, LDL - C, HDL - C, albumin, Tg, TyG - BMI and Gender were significantly different between the two groups (P<0.05), but TPOAb, Total Cholesterol, BG and hypertension were not significantly different between the two groups (P>0.05).

**Table 2:** Clinical characteristics of participants.

Variables	Total (n = 538)	TN (n = 213)	PTC (n = 325)	*P*
Age, M (Q_1_, Q_3_)	47.00 (37.00, 56.00)	51.00 (41.00, 58.00)	44.00 (35.00, 54.00)	**<.001**
Height, M (Q_1_, Q_3_)	162.30 (159.00, 165.75)	161.20 (158.00, 163.80)	163.60 (160.00, 166.60)	**<.001**
Weight, M (Q_1_, Q_3_)	64.20 (60.00, 69.70)	61.80 (58.00, 66.40)	66.00 (61.80, 70.00)	**<.001**
BMI, M (Q_1_, Q_3_)	24.24 (23.20, 25.81)	23.83 (22.64, 25.47)	24.57 (23.51, 26.03)	**<.001**
Leukocyte count (10^9^/L), M (Q_1_, Q_3_)	5.60 (4.62, 6.68)	5.50 (4.60, 6.40)	5.70 (4.70, 6.70)	**0.041**
TgAb (IU/mL), M (Q_1_, Q_3_)	11.87 (6.77, 73.95)	10.50 (6.55, 32.71)	12.64 (7.12, 85.50)	**0.030**
TPOAb (IU/mL), M (Q_1_, Q_3_)	4.70 (2.08, 16.98)	5.10 (2.08, 16.97)	4.46 (2.07, 16.99)	0.844
THG (ng/mL), M (Q_1_, Q_3_)	13.17 (4.61, 34.93)	19.87 (9.25, 62.21)	9.68 (2.80, 26.92)	**<.001**
TSH mIU/L, M (Q_1_, Q_3_)	2.20 (1.50, 3.18)	2.06 (1.38, 2.83)	2.35 (1.59, 3.37)	**0.003**
LDL - C, M (Q_1_, Q_3_)	2.65 (2.24, 3.01)	2.52 (2.18, 2.88)	2.69 (2.34, 3.14)	**0.002**
HDL - C, M (Q_1_, Q_3_)	1.23 (1.07, 1.44)	1.32 (1.12, 1.50)	1.20 (1.04, 1.36)	**<.001**
Total Cholesterol, M (Q_1_, Q_3_)	4.53 (4.09, 5.05)	4.46 (3.99, 4.97)	4.55 (4.14, 5.08)	0.136
Albumin, M (Q_1_, Q_3_)	42.35 (40.00, 45.27)	41.90 (39.70, 45.10)	42.80 (40.30, 45.40)	**0.023**
Tg (mg/dl), M (Q_1_, Q_3_)	103.63 (77.94, 147.03)	93.00 (69.08, 127.54)	113.37 (85.03, 155.00)	**<.001**
BG (mg/dl), M (Q_1_, Q_3_)	90.46 (84.33, 98.21)	90.10 (84.33, 97.31)	90.46 (84.33, 98.39)	0.573
TyG - BMI, M (Q_1_, Q_3_)	206.06 (192.62, 223.21)	201.21 (182.97, 217.76)	208.53 (196.70, 230.06)	**<.001**
Gender, n(%)				**0.034**
Female	395 (73.42)	167 (78.40)	228 (70.15)	
Male	143 (26.58)	46 (21.60)	97 (29.85)	
Hypertension, n(%)				0.576
No	448 (83.27)	175 (82.16)	273 (84.00)	
Yes	90 (16.73)	38 (17.84)	52 (16.00)	

PTC, papillary thyroid carcinoma; TN, thyroid nodule; BMI, body mass index; HDL-c, high-density lipoprotein cholesterol; LDL-c, low-density lipoprotein cholesterol; THG, Thyroglobulin; TgAb, Thyroglobulin Antibody; TPOAb, Thyroid Peroxidase Antibody; TSH, Thyroid-Stimulating Hormone,. Bold values, P<0.05, be statistically different.

**Figure 2:**
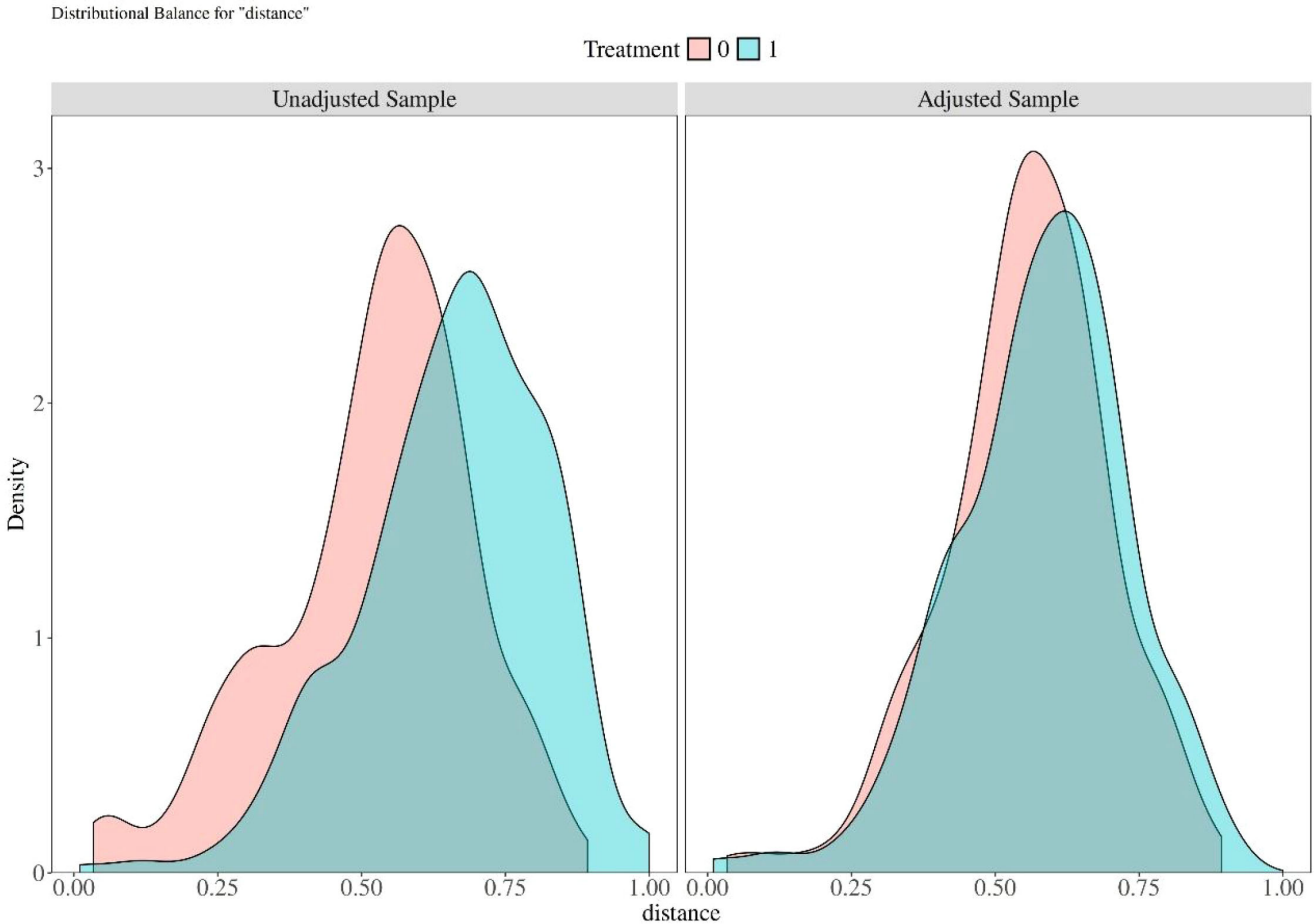
The probability density analysis graphs of the samples prior to and following matching.

To reduce the statistical bias caused by differences in sample sizes between groups, we used propensity score matching (PSM) to balance the inter-group sample sizes. Specifically, the nearest neighbor matching method was adopted, with a caliper value set to 0.2, and matching was performed for all covariates to achieve a 1:1 sample ratio between the two groups (**Table 3**). **Figure 2** presents the probability density analysis graphs of the samples prior to and following matching.

**Table 3:** The baseline table of participants included after propensity score matching (PSM).

Variables	Total (n = 382)	TN (n = 191)	PTC (n = 191)	*P*
Age, M (Q_1_, Q_3_)	49.00 (39.00, 57.00)	49.00 (40.00, 56.50)	48.00 (39.00, 58.00)	0.449
Leukocyte count (10^9^/L), M (Q_1_, Q_3_)	5.50 (4.60, 6.50)	5.40 (4.55, 6.40)	5.50 (4.60, 6.60)	0.541
TgAb (IU/mL), M (Q_1_, Q_3_)	11.52 (6.65, 56.19)	11.44 (6.61, 33.60)	11.74 (6.80, 78.90)	0.409
TPOAb (IU/mL), M (Q_1_, Q_3_)	4.61 (2.08, 14.44)	5.31 (2.09, 16.99)	4.15 (2.04, 12.10)	0.271
THG (ng/mL), M (Q_1_, Q_3_)	2.11 (1.43, 3.03)	2.06 (1.38, 2.84)	2.18 (1.48, 3.09)	0.194
LDL-C, M (Q_1_, Q_3_)	2.58 (2.20, 2.94)	2.56 (2.22, 2.90)	2.59 (2.20, 3.04)	0.469
HDL-C, M (Q_1_, Q_3_)	1.28 (1.12, 1.48)	1.32 (1.12, 1.49)	1.27 (1.12, 1.46)	0.741
Total Cholesterol, M (Q_1_, Q_3_)	4.46 (4.04, 4.99)	4.46 (4.00, 4.97)	4.47 (4.06, 5.00)	0.697
Albumin, M (Q_1_, Q_3_)	42.00 (39.70, 44.70)	41.90 (39.75, 45.10)	42.00 (39.60, 44.65)	0.899
Tg (mg/dl), M (Q_1_, Q_3_)	98.31 (72.63, 132.85)	93.88 (69.08, 127.54)	100.08 (78.38, 137.28)	0.086
BG (mg/dl), M (Q_1_, Q_3_)	90.64 (84.33, 98.16)	90.10 (84.33, 97.58)	91.00 (84.42, 98.39)	0.550
Gender, n(%)				0.520
Female	307 (80.37)	151 (79.06)	156 (81.68)	
Male	75 (19.63)	40 (20.94)	35 (18.32)	
Hypertension, n(%)				1.000
No	318 (83.25)	159 (83.25)	159 (83.25)	
Yes	64 (16.75)	32 (16.75)	32 (16.75)	

### The PTC odds and the TyG - BMI index

**Figure 3:**
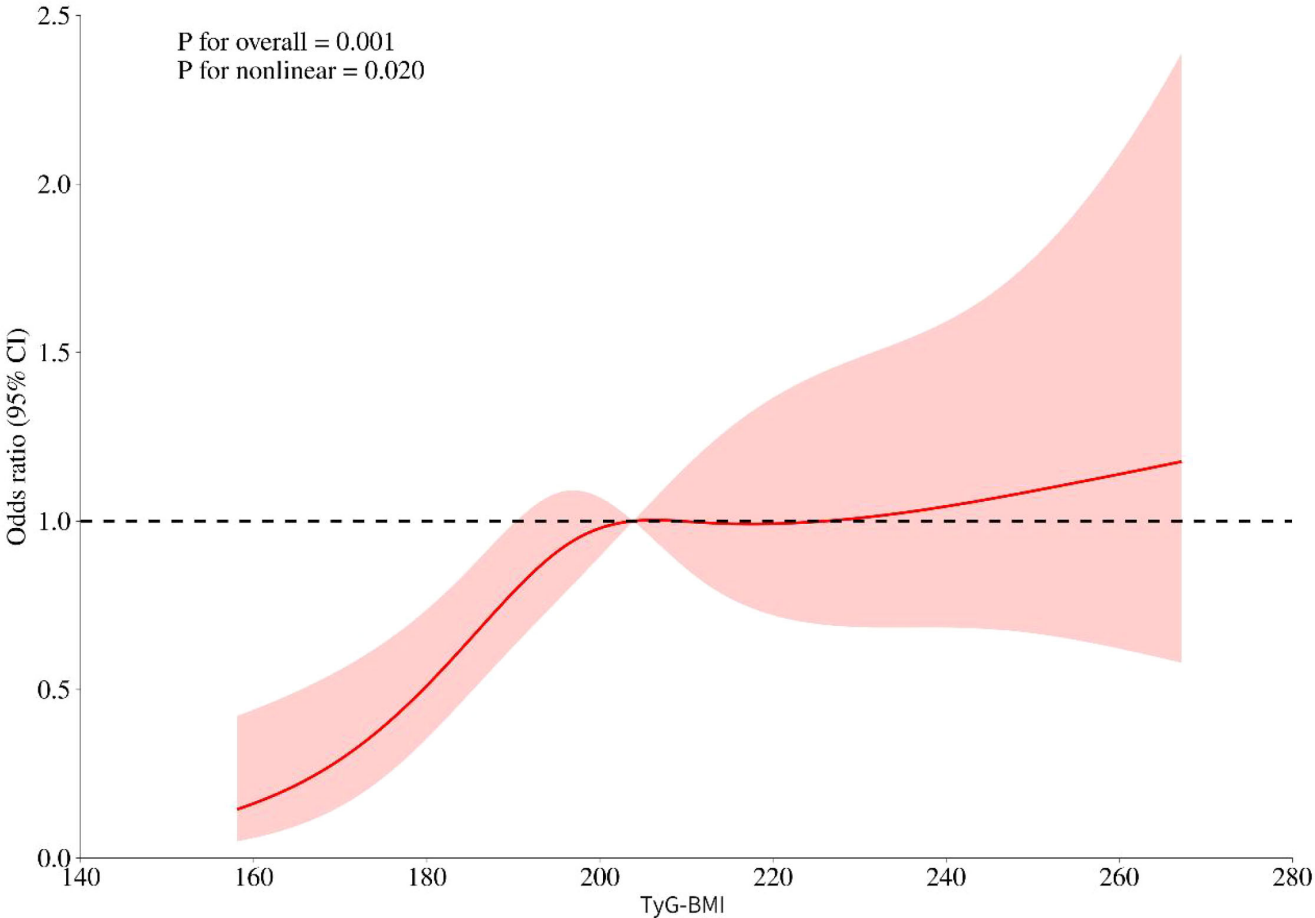
The RCS curve between TyG-BMI and papillary thyroid carcinoma.

**Figure 3** shows the link between the odds of papillary thyroid carcinoma (PTC) and the TyG - BMI index. In **Table 4**, we divided TyG - BMI into high-level and low-level groups based on the level of median value. Then we used three models to assess the relationship between TyG - BMI and papillary thyroid carcinoma. Model 1 was unadjusted. Model 2 was adjusted for age, sex. Model 3 was further adjusted for total cholesterol, albumin, leukocyte count, high - density lipoprotein cholesterol, low - density lipoprotein cholesterol, Tg, TgAb, TPOAb, TSH and hypertension based on Model 2.

**Table 4:** Analysis of the odds relationship between TyG-BMI and papillary thyroid carcinoma.

TyG-BMI	Model 1 (OR,95%CI)	P	Model 2 (OR,95%CI)	P	Model 3 (OR,95%CI)	P
Continuous	1.01(1.01-1.02)	0.001	1.01(1.01-1.02)	<0.001	1.02(1.01-1.03)	<0.001
<206.11	1(Reference)	——	1(Reference)	——	1(Reference)	——
≥206.11	1.59(1.06-2.39)	0.024	1.67(1.10-2.51)	0.015	1.79(1.13-2.83)	0.013

Model 1, unadjusted. Model 2, adjusted for age, sex. Model 3, Model 2+ total cholesterol, albumin, leukocyte count, high-density lipoprotein cholesterol, low-density lipoprotein cholesterol, Tg, TgAb, TPOAb, TSH and hypertension.

The results showed (**Figure 3**) a non-linear relationship between TyG - BMI and the odds of papillary thyroid carcinoma (PTC). In Model 1, for each 1-unit increase in TyG - BMI, the odds ratio (OR) of papillary thyroid carcinoma (PTC) was approximately 1.01(P = 0.001). With the lowest group as the reference, the OR of the highest group was 1.59 (95% CI, 1.06-2.39, P = 0.024). In Model 2, for each 1-unit increase in TyG - BMI, the odds ratio (OR) of papillary thyroid carcinoma (PTC) was approximately 1.01 (P<0.001). With the lowest group as the reference, the OR of the highest group was 1.67 (95% CI, 1.10 - 2.51, P = 0.015). In Model 3, for each 1-unit increase in TyG-BMI, the odds ratio (OR) of papillary thyroid carcinoma (PTC) was approximately 1.02 (P<0.001). With the lowest quantile group as the reference, the OR of the highest quantile group was 1.79 (95% CI, 1.13 - 2.83, P = 0.013). Furthermore, to control the family-wise error rate (FWER) of the statistical results, we performed the Holm-Bonferroni correction on the statistical outcomes of the three models. With the significance threshold α set at 0.05, the adjusted p-values of all the three models were statistically significant.

### The efficacy of TyG-BMI in the diagnosis of papillary thyroid carcinoma

**Figure 4:**
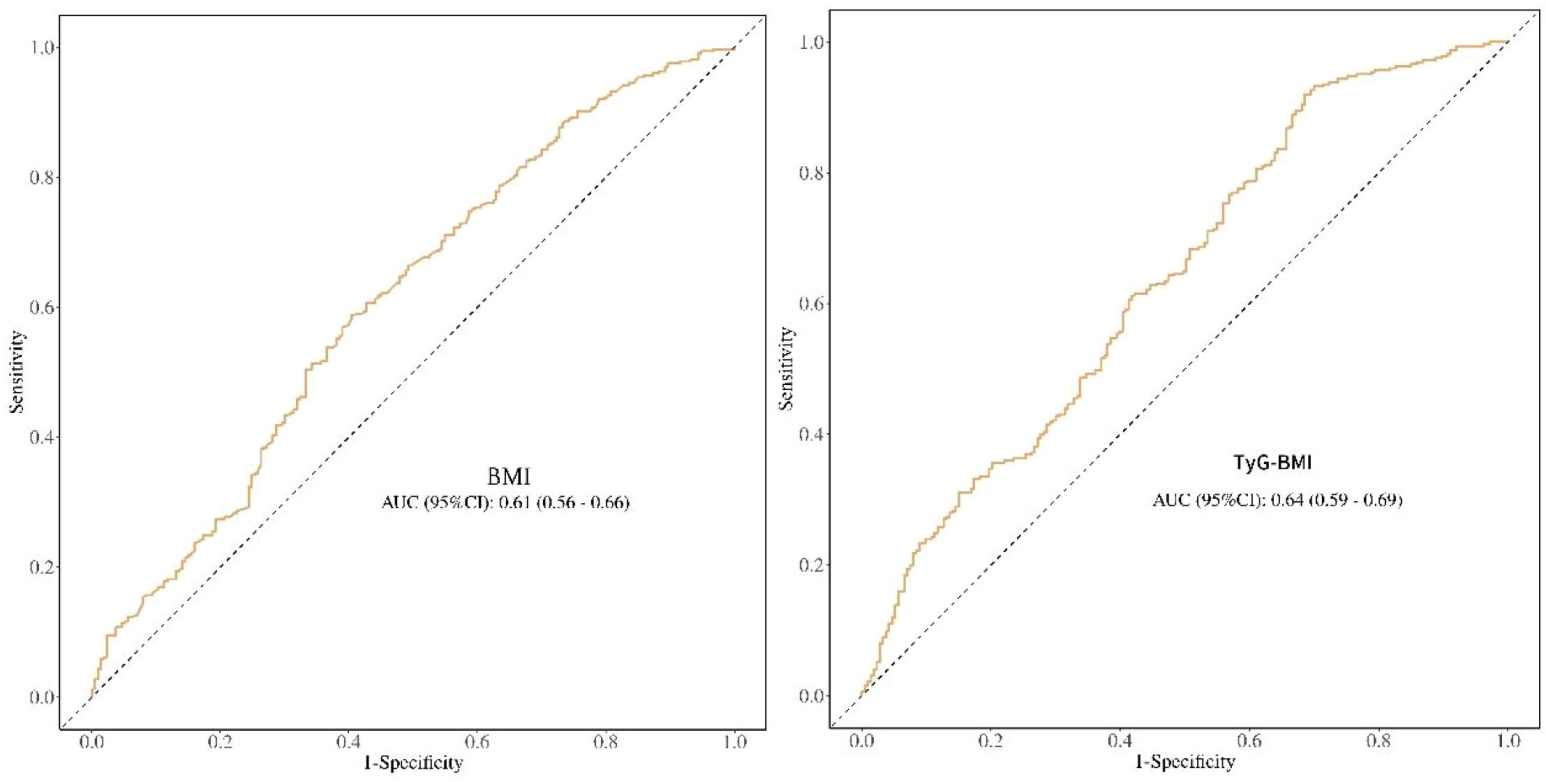
The ROC curve of BMI and Tyg-BMI for the diagnosis of papillary thyroid carcinoma.

To assess the efficacy of triglyceride glucose-body mass index (TyG-BMI) in diagnosing papillary thyroid carcinoma (PTC), we conducted a receiver operating characteristic (ROC) curve analysis. The results (**Figure 4**) showed that the diagnostic efficacy of TyG-BMI for papillary thyroid carcinoma was relatively low (AUC = 0.64), yet it was improved compared with that of body mass index (BMI) in diagnosing papillary thyroid carcinoma (AUC = 0.61). In the future, it will be necessary to further explore and enhance the efficacy of TyG-BMI in diagnosing papillary thyroid carcinoma (PTC).

## Discussion

This study aims to thoroughly explore the connection between the TyG - BMI index and papillary thyroid carcinoma. It includes 213 patients with benign thyroid nodule and 325 patients with papillary thyroid carcinoma, constituting a comprehensive examination of the relationship between the TyG - BMI index and papillary thyroid carcinoma among Chinese residents. The TyG - BMI index of papillary thyroid carcinoma (PTC) patients is notably higher than that of patients with thyroid nodule patients. Interestingly, higher levels of the TyG - BMI index are linked to a greater odds of advanced papillary thyroid carcinoma. This suggests that TyG - BMI could be a potentially accessible indicator associated with papillary thyroid carcinoma odds.

An increasing amount of epidemiological data indicates a connection between metabolic syndrome and the occurrence of papillary thyroid carcinoma. Furthermore, this correlation is related to a worse response to treatment, a quicker pace of disease progression, and a less optimistic prognosis (22). Insulin resistance involves reduced liver glucose production and decreased sensitivity to insulin’s ability to help cells take up glucose (23). This makes a significant contribution to the pathogenesis of MetS (24, 25). Since insulin resistance is key in developing metabolic syndrome, it’s useful to see how well the TyG - BMI index can spot MetS (14, 26). The TyG - BMI index is a reliable marker for MetS, showing a high sensitivity and specificity (27).

Although our observational data show an association between TyG - BMI and PTC odds, the underlying biological mechanisms remain unknown and what follows is purely theoretical speculation. Based on existing literature, we hypothesize the following plausible but entirely unverified pathways as a framework for future research. TyG - BMI is associated with insulin resistance, which may theoretically disrupt the PI3K/AKT signaling and oxidative stress pathways in thyroid tissue. However, whether these pathways specifically act on PTC and whether there are differences among different molecular subtypes (such as BRAF mutant vs. wild type) could not be observed in our study, and further experimental exploration is needed in the future.

One hypothetical mechanism involves insulin signaling disruption. In theory, insulin receptor binding might activate downstream molecules including IRS phosphorylation and subsequent PI3K/AKT pathway activation (28). In the context of insulin resistance, this pathway could potentially become dysregulated, theoretically affecting cell proliferation and survival. For instance, insulin resistance might alter IRS phosphorylation patterns, potentially impairing PI3K binding and AKT activity. These speculated events, if they occur in thyroid tissue, could theoretically be associated with abnormal cellular proliferation (29). It must be emphasized that no data from this study support this specific sequence of events in PTC.

Another theoretical consideration involves oxidative stress. Hyperinsulinemia and inflammation have been speculatively linked to oxidative stress in non-thyroid experimental systems. If applicable to thyroid tissue, insulin resistance might impair mitochondrial function and electron transport, potentially increasing ROS production (30). Inflammatory factors could theoretically activate redox enzymes such as NADPH oxidase. While ROS can damage cellular macromolecules including DNA, whether this specifically initiates PTC-related mutations (e.g., BRAF, RET) in humans is unknown (31). This study did not measure oxidative stress markers, ROS levels, or DNA damage in any tissue, and these proposed connections remain entirely speculative. Currently, research on how metabolic syndrome (MetS) induces papillary thyroid carcinoma (PTC) remains limited, and further investigation in this regard is required in the future.

### Critical limitations of mechanistic interpretation

The pathways discussed above suffer from significant limitations: (1) They are extrapolated from general metabolic and cancer biology literature, not PTC-specific research; (2) No molecular, cellular, or tissue-level data were collected in this study; (3) The potential heterogeneity of PTC (e.g., BRAF-mutant vs. RAS-mutant vs. wild-type tumors) was not addressed; (4) Temporal relationships cannot be established from our cross-sectional design. These mechanisms are presented solely as hypothesis-generating frameworks requiring validation in mechanistic studies incorporating PTC molecular subtyping and functional assays.

## Conclusion

Overall, this study found that the TyG-BMI index is associated with the odds of papillary thyroid carcinoma. However, due to the limitations of the current retrospective study, its potential utility as a tool for predicting papillary thyroid carcinoma risk remains to be explored in larger-scale and more rigorous studies in the future.

### Study limitations

First, there was a lack of detailed information on important factors like fasting time, medication use, lifestyle, dietary habits and exercise levels, which might have influenced the results. Secondly, the study was limited to a single-center sample, which may restrict the generalizability of the results. Due to data limitations, some factors related to PTC cannot be considered, such as radiation exposure, iodine intake, family history of thyroid cancer, nodule size, ultrasound risk characteristics, or cytological categories, and tumor invasiveness, which could potentially affect the validity of our conclusions. So our conclusions should not be over-interpreted and more rigorous cohort design is needed in the future The control group (Thyroid nodules) is not metabolically neutral and may be related to metabolic abnormalities, which limits the interpretation of TyG - BMI as a marker for PTC in the general population and prevents our conclusions from being effectively extrapolated to healthy populations. Lastly, the retrospective study design made it impossible to prove the causal relationship between TyG - BMI and papillary thyroid carcinoma (PTC), because we can’t establish the sequence of exposure and causation.

## Data Availability

The raw data supporting the conclusions of this article will be made available by the authors, without undue reservation.
